# COVID-19 Infection in Pediatric Patients Presenting to a Tertiary Center in Jordan: Clinical Characteristics and Age-Related Patterns

**DOI:** 10.3390/jcm14082577

**Published:** 2025-04-09

**Authors:** Montaha Al-Iede, Marah Dannoun, Iyad Al-Ammouri, Enas Al-Zayadneh, Amirah Daher, Jumana Albaramki, Danah Alsmady, Omar Husain, Batool Abusabra, Rima A. Sinan, Lena Sarhan

**Affiliations:** 1 Division of Pediatric Pulmonology and Sleep Medicine, Department of Pediatrics, Jordan University Hospital, Amman 11942, Jordan; marahldan@gmail.com (M.D.); e.alzayadneh@ju.edu.jo (E.A.-Z.); danah.moath94@gmail.com (D.A.); dromarfahim@gmail.com (O.H.); batoolabosabrah@gmail.com (B.A.); reemaaltatar98@gmail.com (R.A.S.); lenasarhan@gmail.com (L.S.); 2 The School of Medicine, The University of Jordan, Amman 11942, Jordan; a.taher@ju.edu.jo (A.D.); j.albaramki@ju.edu.jo (J.A.); 3 Division of Cardiology, Department of Pediatrics, Jordan University Hospital, Amman 11942, Jordan; i.ammouri@ju.edu.jo; 4 Division of Pediatric Intensive Care, Department of Pediatrics, Jordan University Hospital, Amman 11942, Jordan; 5 Division of Pediatric Nephrology, Department of Pediatrics, Jordan University Hospital, Amman 11942, Jordan

**Keywords:** pandemic, outcome, COVID-19, symptoms, management

## Abstract

**Objectives:** This study assessed the clinical characteristics of pediatric COVID-19 patients across different age groups during the first and second pandemic waves in Jordan. **Methods:** A retrospective analysis was conducted at Jordan University Hospital, involving 485 patients aged 1 month to 18 years from September 2020 to July 2021. Patients were categorized into preschool (≤5 years), school-aged (6–10 years), and teenagers (>10 years). Patients’ clinical characteristics were analyzed using R (version 2.3.3). **Results:** The mean age for participants was 10.7 ± 5.7 years. Shortness of breath, abdominal pain, and headaches were significantly more likely among older participants (all *p* < 0.01). Conversely, younger patients were more likely to experience nasal congestion, decreased activity, and reduced feeding (all *p* < 0.05). The majority of patients had mild symptom severity. Analysis of physiologic and laboratory parameters demonstrated significant differences among age groups in terms of heart rate, respiratory rate, hemoglobin, neutrophils, lymphocytes, platelets, CRP, and creatinine (all *p* < 0.05). Respiratory support was mainly observed among younger patients. Antibiotics was the most commonly received medication. In terms of outcomes, two patients had complications during their stay, both of which belonged to the <5 years age group. We observed significant differences in incidence of symptoms and laboratory markers among different pediatric age groups. While younger patients experienced severe complications, their older counterparts exhibited more alarming symptoms and worse counts of immune cells. **Conclusions:** These findings highlight the importance of age-specific management strategies for COVID-19, emphasizing the need for tailored approaches in both treatment and prevention.

## 1. Introduction

The coronavirus disease 2019 (COVID-19) pandemic has significantly affected the lives of many worldwide. In Jordan, widespread community transmission began in September and peaked in November 2020 as the initial COVID-19 wave [1]. The swift propagation of the UK variant contributed to a more aggressive second wave in January through to March 2021 [2]. Nearly three years later, the virus continues to cause unprecedented global morbidity, mortality, and disruption, supported by numerous studies reflecting diverse impacts related to factors such as disease onset, symptoms, complications, and SARS-CoV-2 variants [3]. However, as the health crisis becomes less threatening, the impact of the disease is far from over.

When it comes to COVID-19 infections, the literature indicates that pediatric populations exhibit lower frequency and reduced severity, with approximately 5% of cases and a notably low fatality rate compared to adults [4,5,6]. However, a plethora of studies found that the incidence rate of SARS-CoV-2 infections in children was similar to adults, although most were asymptomatic [7]. However, pediatric patients with complex chronic conditions and younger age are at higher risk for severe illness and more serious complications [8]. Such complications include acute respiratory distress syndrome, myocarditis, acute renal failure, multisystem organ failure, and multisystem inflammatory syndrome in children (MIS-C) [9].

While most pediatric patients remain asymptomatic, patients that demonstrate symptoms have a nonspecific presentation. Such nonspecific symptoms include fever, cough, sore throat, myalgia, headache, and malaise. Moreover, gastrointestinal symptoms, such as abdominal pain, diarrhea, and vomiting, are also frequently observed [10]. While olfactory dysfunction—manifesting as loss of smell or taste—was initially primarily considered a feature in adults, recent studies have demonstrated its notable prevalence in pediatric cases as well [11]. Finally, while neurological symptoms are commonly reported, they are mostly transient with limited pathological involvement [12]. Nonetheless, life-threatening conditions, such as stroke, encephalopathy, and cerebral edema, may still occur in a small proportion of patients.

Up until the writing of this manuscript, the mainstay management of COVID-19 is supportive care [10], particularly within low-to-middle-income settings. Patients with high-risk conditions can benefit from antiviral therapy (e.g., nirmatrelvir–ritonavir, remdesivir) within 5–7 days of symptom onset. Moreover, in patients who are hospitalized and require oxygen, dexamethasone can also be considered [13].

Unfortunately, despite the gravity of the COVID-19 pandemic, research on its effects on pediatric patients generally in the Middle East, particularly in Jordan, is lacking. In fact, most research is directed at knowledge of either physicians or patients towards the disease or is primarily concerned with adult-only cohorts. Therefore, this study provides a comprehensive overview of pediatric patients in different age groups during the initial and second waves of the pandemic in Jordan.

## 2. Materials and Methods

### 2.1. Study Design, Patients, and Clinical Data

This retrospective observational study was conducted at Jordan University Hospital (JUH) to evaluate COVID-19 infections among pediatric patients in Jordan. Data were collected from patients aged 1 month to 18 years who tested positive for SARS-CoV-2 from September 2020 to July 2021 during the first and second waves of the pandemic. The first wave of COVID-19 encompassed the period between November 2020 and March 2021, while the second wave included the period between March 2021 and January 2022 [14]. The first wave was dominated by the B.1.1.312, B.1.36.10 Jordanian variants and the B.1.1.7 UK variant [2]. In addition to the aforementioned, the second wave witnessed a spike in the “Delta Plus” variant among diagnosed cases.

### 2.2. Inclusion and Exclusion Criteria

Nasopharyngeal aspirates (NPA) were used to detect the SARS-CoV-2 virus using conventional RT-PCR. The reason for testing included symptom complaints, contact screening, and routine screening before surgeries or travel. A total of 485 patients were included in the final analysis. The exclusion criteria for this study were as follows: individuals with immunosuppression, including transplant recipients; patients with cancer or those receiving immunosuppressive therapies or chemotherapy; individuals with other chronic conditions or medications that could influence the clinical presentation; cases of prolonged COVID-19 or MIS-C; co-infections.

### 2.3. Data Collection and Measures

The medical records of 485 patients with SARS-CoV-2-positive nasopharyngeal aspirate (NPA) results were reviewed. Patients were categorized into three age groups: preschool (≤5 years), school-aged (6 to 10 years), and teenagers (>10 years). Age classification and grouping was adapted from the WHO/UNICEF and NICHD guidelines. Data were obtained from both medical records and caregivers in an attempt to reduce missing information. Collected data included symptomatology profiles and COVID-19 infection history. Information regarding vital signs and laboratory results were obtained from patient records. Laboratory measures included hemoglobin (6 to 60 months, >100 g/L; 6 to 11 years, >115 g/L; 12 to 18 years, >120 g/L), WBC (4 to 10 cell/L), neutrophils (40–80%), lymphocytes (20–40%), platelets (150–450 cell/L), CRP (>5 mg/L), ESR (0–10 mm/h), creatinine (0.3–1 mg/dL), AST (10–14 U/L), ALT (5–55 U/L), GGT (5–25 U/L), and ALP (70–300 U/L).

Additionally, details regarding the patient’s clinical course during hospitalization were gathered, including medication use and respiratory support interventions. Oxygen delivery methods included low-flow nasal cannula (LFNC), high-flow nasal cannula (HFNC), and positive pressure ventilation (PPV). The severity of COVID-19 symptoms was classified as asymptomatic, mild, moderate, or severe, in accordance with WHO guidelines from the corresponding period, as shown in Table 1 [15].

COVID-19 death, defined as death resulting from a clinically compatible illness in a probable or confirmed COVID-19 case unless an alternative cause of death not related to COVID-19 disease (e.g., trauma), was identified [15]. When in doubt, the cause of mortality to be assigned was discussed and reviewed by the clinician in-charge and COVID-19 Committee Chair. Other clinical outcomes included the development of complications during acute infection such as bacteremia, stroke, arrhythmia, barotrauma, pulmonary embolism, deep vein thrombosis, or myocarditis.

### 2.4. Nasopharyngeal Sampling and COVID-19 Detection

The results of multiplex respiratory pathogen real-time PCR tests for nasopharyngeal swab specimens were extracted from the electronic-based molecular diagnostic laboratory record of JUH. Nasopharyngeal swabs were collected using the universal transport and preservation of Copan media and stored at −20 °C. Conventional real-time PCR, specifically the FTD Respiratory Pathogens 21 Assay Kit and EZ 1 and 2 Virus Mini Kit V2.0 by Qiagen (Hilden, Germany), was employed for nucleic acid extraction. The viruses detected in this study included SARS-CoV-2, human adenovirus, human rhinovirus and enterovirus, human parainfluenza viruses 1–4, respiratory syncytial virus (RSV), metapneumovirus A and B (MNPV), influenza A, A/H1N1, and B, as well as coronaviruses 229E, NL63, OC43, and HKU1.

### 2.5. Statistical Analysis

Data were imported in and analyzed using R (version 2.3.3). Continuous variables were summarized as mean and standard deviation, while categorical variables were summarized as counts and percentages. Associations between categorical variables were assessed using the chi-squared test. Differences in continuous variables were assessed using the Kruskal–Wallis test and associated post hoc testing with Bonferroni correction for multiple comparisons. A *p*-value of <0.05 was considered statistically significant.

### 2.6. Ethics Approval

The study was conducted according to the guidelines of the Declaration of Helsinki and approved by the Institutional Review Board of JUH (2021/109: 24 March 2021). In addition, this study adheres to the Strengthening the Reporting of Observational Studies in Epidemiology (STROBE) guidelines. Informed consent from patients was not required due to the retrospective nature of the study, as per the guidelines of our ethics committee.

## 3. Results

### 3.1. Demographics and Clinical Presentation of the Patients

**Figure 1:**
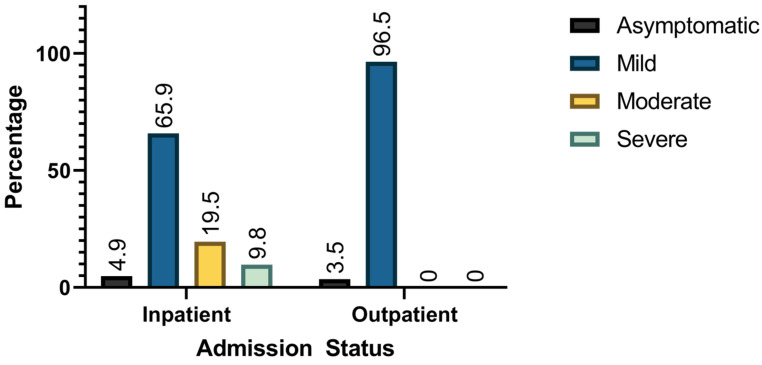
COVID-19 severity stratified by admission status.

**Figure 2:**
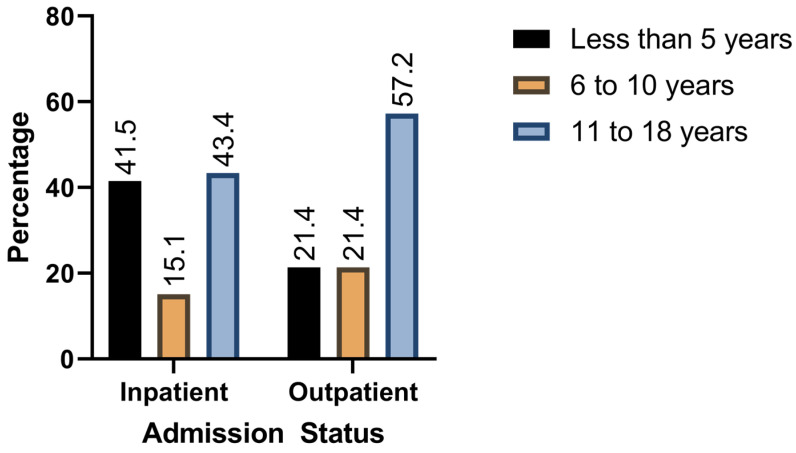
Age groups stratified by admission status.

The mean age of included patients was 10.7 ± 5.7 years. Of the included participants, 49.5% were males, while 45.2% were females. About 11.5% of patients were admitted, whereas 88.5% were treated as outpatients. The median duration of hospital stay for included patients was 1 day (1–24). Among admitted patients, 4.9% were asymptomatic, 65.9% had a mild presentation, 19.5% had a moderate presentation, and 9.8% had a severe presentation. On the other hand, mild (96.5%) and asymptomatic (3.5%) were the only presentations among outpatients. Figure 1 and Figure 2 demonstrates admission status per age group and COVID-19 severity.

In terms of symptoms, fever (52.7%), cough (36.6%), sore throat (19.3%), rhinorrhea (18.7%), and headache (18.2%) were the most common among the included participants. Conversely, seizures (3.3%), insomnia (3.3%), loss of taste (2.2%), skin rash (2.2%), and conjunctivitis (1.1%) were the rarest. Table 2 highlights the associations between symptomatology and age. Participants in the older age group were significantly more likely to experience shortness of breath (*p* = 0.005), myalgia (*p* < 0.001), abdominal pain (*p* = 0.010), and headaches (*p* < 0.001). On the other hand, younger participants were more likely to have nasal congestion (*p* = 0.032), decreased activity (*p* = 0.005), and reduced feeding (*p* = 0.003). Across all age groups, the majority of participants had mild symptom severity.

### 3.2. Physiologic and Laboratory Parameters

Analysis of physiologic and laboratory parameters demonstrated significant differences among age groups in terms of heart rate (*p* < 0.001), respiratory rate (*p* < 0.001), hemoglobin (*p* = 0.008), neutrophils (*p* < 0.001), lymphocytes (*p* < 0.001), platelets (*p* = 0.019), CRP (*p* = 0.033), and creatinine (*p* < 0.001) (refer to Table 3).

In post hoc testing, older patients (i.e., older than 11 years) had significantly lower mean heart rate and respiratory rate compared to both the <5 and 6-to-10-year age groups. Similarly, patients in the 6-to-10-year age group had a significantly lower respiratory rate than their <5 years counterparts.

In terms of hematology and biochemistry markers, older patients had significantly higher mean hemoglobin, neutrophils, and creatinine compared to their counterparts in the <5 years age group. Conversely, patients in the <5 years age group had significantly higher mean lymphocytes and platelet counts. Supplementary Table S1 demonstrates the categorized hematological and biochemical profiles of patients stratified by age.

### 3.3. Management and Outcome

Table 4 illustrates the respiratory support modalities, medications, and outcomes among included participants. Patients within the <5 years age group were primarily the cohort which received respiratory support, mainly O2 support. While none of the included cohort received anti-viral therapy, 62 participants received antibiotics, 12 received steroids, and only 1 patient received inotropes. In terms of outcomes, two patients had complications during their stay (i.e., mortality), both of which belonged to the <5 years age group and were designated as severe cases, as per WHO guidelines.

## 4. Discussion

Symptoms such as fever, cough, and shortness of breath are common in pediatric COVID-19 cases, while headaches and seizures appear less frequently. Certain symptoms, like myalgia and headache, were significantly associated with different age groups. Compared to adults, children with COVID-19 exhibit lower rates of fever but similar occurrences of cough, myalgia, and sore throat [16,17]. Diarrhea rates are comparable, but vomiting is more prevalent in children [18].

Pediatric SARS-CoV-2 infection can present with neurological signs and symptoms, including headache, irritability, lethargy, and myalgia indicating potential neuromuscular involvement [19]. SARS-CoV-2 RNA has been detected in cerebrospinal fluid, suggesting viral activity in the central nervous system [20]. Other mechanisms such as hypoxic injury and immune response may also contribute to neurologic manifestations associated with COVID-19 [20]. While neurologic symptoms in younger children may be underreported due to communication challenges, physicians and pediatricians should consider COVID-19 as a differential diagnosis for such symptoms. In our study, headaches were significantly more reported by or observed among the oldest cohort. While this difference may be related to the ability of older patients to communicate their patients more effectively, a South Korean study observed that neurological symptoms (e.g., febrile seizures) do have a predilection towards older patients and male gender [21].

Skin changes, though rare in this study, can occur with COVID-19, possibly due to immune responses or systemic effects like vasculitis [22]. While loss of smell (anosmia) and taste (ageusia) are common symptoms in adults, they are less frequent in children [23]. This aligns with our study which had a low prevalence of anosmia and ageusia, which could be due to challenge of expressing the absence of smell and taste in children. This could be the case as all of the patients who had those symptoms were over the age of 11. The mechanism of SARS-CoV-2 ageusia remains speculative, possibly involving coronaviruses affecting taste buds’ sialic acid binding sites. Children’s lower ACE2 expression in oral and nasal mucosa might extend to non-nervous olfactory tissue [24], possibly explaining the lower prevalence of anosmia in children.

The severity categorization in this study indicated that the majority of patients experienced mild clinical forms of COVID-19. Age is a risk factor in pediatric COVID-19 severity, with infants, particularly those younger than one year, being vulnerable [4]. In our findings, severe presentation was observed in the youngest and oldest age group. Some studies suggest a bimodal distribution of severe pediatric COVID-19 cases, affecting infants and adolescents [10]. Children with underlying health conditions across age groups are also at heightened risk [10]. Notably, our study excluded patients with underlying conditions, potentially contributing to the observed milder presentations of COVID-19 in our cohort.

In terms of vital signs, the youngest cohort had significantly higher mean heart and respiratory rates compared to their oldest counterparts. Most infants, children, and adolescents with COVID-19 recover fully without lasting cardiac dysfunction, but further research is needed to assess cardiovascular risks with SARS-CoV-2 variants and understand the associated cardiac dysfunction’s pathophysiology [25].

On the other hand, hematological parameters within our study, including hemoglobin levels, platelet count, and white blood cell count, were within typical pediatric ranges, suggesting a relatively normal hematological profile. Elevated inflammatory markers such as CRP and ESR reflect expected inflammatory responses. Hemoglobin levels vary significantly among age groups, with the oldest cohort having the highest mean Hb. This variation could be attributed to age-related disparities in hematological parameters, influencing oxygen-carrying capacity and potentially impacting disease severity. Platelet counts also differ across age groups, emphasizing potential age-related disparities in clotting mechanisms. The higher mean platelet counts in the oldest cohort suggest age-specific variations in hemostasis, warranting further investigation into the thrombotic aspects of COVID-19 in different age demographics.

Studies among adults show that males with COVID-19 had higher neutrophils, CRP, and inflammatory cytokine levels, along with lower lymphocyte levels, which are associated with poor prognosis [26]. However, lymphocytopenia, a crucial feature in predicting adult COVID-19 severity, is rare in children [27]. Weak immune responses in children were indicated by less frequent reports of leukopenia, high CRP levels, high erythrocyte sedimentation rate, and high ALT levels [28]. Another study found significant relationships between WBC and RBC parameters [29]. In our study, older patients had significantly lower mean WBC and lymphocyte counts, suggesting an impact on the immune response.

Biochemical markers offer further insights into liver function. Age-stratified analysis showed intriguing patterns: ALT levels increased with age, peaking in the oldest cohort, while GGT levels decreased with age. These trends suggest potential developmental influences on liver function parameters in pediatric patients, though larger sample sizes are needed for confirmation. Liver injury is an extrapulmonary manifestation of COVID-19, mainly in severe cases [28]. Elevated ALT and AST levels are crucial markers for assessing liver damage in COVID-19 patients [30].

Creatinine levels varied amongst age groups, with the oldest cohort (11 years and above) showing the highest mean creatinine. Increased creatinine is a sign of acute kidney injury. AKI has been found to be associated with MIS-C, a complication of COVID-19 [31]. While AKI may not be highly prevalent in pediatric COVID-19 patients, its presence can be a primary cause of critical illness, often necessitating PICU admission [19].

Notably, while the majority of the cohort did not require respiratory support, the use of low-flow nasal cannula (LFNC), high-flow nasal canula (HFNC), and positive pressure ventilation (PPV) were predominantly seen in the younger age group. Younger children are more susceptible to severe symptoms, necessitating respiratory support. Those requiring such assistance typically experience prolonged hospital stays [32].

Regarding medication use, the majority of patients across all age groups did not require steroids. The effectiveness of systemic corticosteroids in treating hospitalized COVID-19 patients without supplementary oxygen remains uncertain. Studies have shown that there are potential benefits of early methylprednisolone use in moderate-to-severe cases [33]. Similarly, none of the included cohort required antiviral therapies. Recent studies caution against the routine use of remdesivir, even in adults, as the RECOVERY trial demonstrated its ability to reduce ICU stay but not mortality [34]. Another study found that 89% of patients did not receive antiviral drugs, leading to a longer length of hospital stay compared to those who received remdesivir therapy [32].

Finally, the observed trends towards poorer outcomes in younger pediatric age groups emphasize the significant role of age in influencing the severity and progression of COVID-19 in children. Although uncommon, death can occur in the setting of COVID-19 in children. Mortality has been seen to be associated with cardiac and pulmonary comorbidities, hypoxemia, and lower respiratory tract symptoms [35].

There are limitations to this study that must be considered. The retrospective nature of the study design might introduce inherent biases, recall issues, and missing data. Additionally, the reliance on data from a specific healthcare setting may introduce selection bias, potentially limiting the study’s ability to capture a comprehensive view of pediatric COVID-19 cases. Furthermore, the study was unable to differentiate between different sources of COVID-19 infection. Finally, due to sample size limitations and limited number of observed outcomes, analyzing clinical outcomes per stage was not feasible. Larger, multicenter studies would be beneficial for obtaining more representative and robust insights into pediatric COVID-19 dynamics. Despite attempts to control for various factors, unmeasured confounders, such as socioeconomic status, underlying health conditions, and individual variations in immune responses, may still influence the observed associations.

## 5. Conclusions

This study provides a comprehensive analysis of COVID-19 infection among pediatric patients in Jordan. The results reveal that, while the majority of cases were mild, younger children required more therapeutic modalities compared to their older counterparts. In contrast, older children exhibited more alarming symptoms. These findings highlight the importance of age-specific management strategies for COVID-19, emphasizing the need for tailored approaches in both treatment and prevention. Overall, this research offers valuable insights into the differential impact of COVID-19 across pediatric age groups, contributing to the development of more effective strategies to mitigate the disease’s effects on children.

## Figures and Tables

**Table 1:** Classification of COVID-19 symptom severity according to WHO guidelines.

Case	Definition
Asymptomatic	A confirmed case (nasopharyngeal RT-PCR is positive for SARS CoV2) having no clinical signs and symptoms.
Mild	A confirmed case with non-specific upper respiratory tract infections (low-grade fever, runny nose, cough) with no radiological signs of pneumonia and oxygen support (oxygen saturation ≥ 94%).
Moderate	A confirmed case with fever and cough/difficulty in breathing without any danger signs; having the radiological evidence of pneumonia requiring hospitalization with or without the need of oxygen support.(oxygen saturation 90%).
Severe	A confirmed case with fever and cough/difficulty in breathing having at least one danger sign (e.g., inability to breastfeed or drink, lethargy, unconsciousness, or convulsions), together with radiological evidence of pneumonia AND/OR sepsis/septic shock, respiratory failure/ARDS, multiple organ dysfunction (MOD).

**Table 2:** Symptomatology stratified by age group.

Characteristic	Less than 5 Years	6 to 10 Years	11 to 18 Years	*p*-Value ^2^
	N = 112 ^1^	N = 95 ^1^	N = 278 ^1^	
Fever	37 (62%)	24 (57%)	46 (46%)	0.11
Sore throat	7 (12%)	6 (15%)	24 (26%)	0.067
Cough	26 (43%)	11 (27%)	34 (37%)	0.3
Shortness of breath	5 (8.5%)	2 (4.9%)	21 (24%)	0.005
Myalgia	0 (0%)	6 (15%)	18 (20%)	<0.001
Nasal congestion	15 (25%)	4 (10%)	9 (11%)	0.032
Rhinorrhea	15 (25%)	8 (20%)	12 (14%)	0.2
Conjunctivitis	0 (0%)	2 (4.9%)	0 (0%)	NA *
Abdominal pain	2 (3.4%)	5 (12%)	18 (21%)	0.01
Diarrhea	12 (20%)	3 (7.3%)	8 (9.4%)	0.079
Decreased activity	14 (24%)	4 (9.8%)	5 (6.0%)	0.005
Anosmia	0 (0%)	0 (0%)	6 (7.2%)	NA *
Loss of taste	0 (0%)	0 (0%)	4 (4.9%)	NA *
Seizure	1 (1.7%)	1 (2.4%)	4 (4.8%)	NA *
Headache	0 (0%)	5 (12%)	29 (33%)	<0.001
Reduced feeding	16 (28%)	2 (5.0%)	9 (11%)	0.003
Vomiting	12 (21%)	8 (20%)	13 (15%)	0.6
Skin rash	0 (0%)	1 (2.4%)	3 (3.6%)	NA *
Severity of symptoms				>0.9
Asymptomatic	2 (3.7%)	1 (2.9%)	4 (4.3%)	
Mild	47 (87%)	33 (94%)	84 (89%)	
Moderate	3 (5.6%)	1 (2.9%)	4 (4.3%)	
Severe	2 (3.7%)	0 (0%)	2 (2.1%)	

^1^ n (%); ^2^ Pearson’s chi-squared test; Fisher’s exact test. * *p*-values with NA were not produced due to the presence of empty cells.

**Table 3:** Vital signs, hematological profile, and biochemistry profile for included participants stratified by age.

Characteristic	Total	Less than 5 Years	6 to 10 Years	11 to 18 Years	*p*-Value ^2^
	N = 485 ^1^	N = 112 ^1^	N = 95 ^1^	N = 278 ^1^	
		Vital Signs			
Heart Rate	105.29 ± 20.85	117.94 ± 19.56	109.31 ± 20.93	97.47 ± 17.87	<0.001
Respiratory Rate	24.20 ± 9.15	31.84 ± 13.69	22.46 ± 5.02	20.84 ± 2.93	<0.001
O_2_ Saturation	96.93 ± 6.70	95.30 ± 11.86	97.79 ± 1.29	97.49 ± 2.88	0.3
Temperature	37.37 ± 2.94	37.04 ± 0.68	38.74 ± 6.79	37.09 ± 0.75	0.054
		Hematological Profile			
Hemoglobin	12.94 ± 1.92	12.20 ± 2.31	12.64 ± 1.47	13.48 ± 1.56	0.008
WBC	8.92 ± 4.13	9.78 ± 4.13	8.40 ± 3.63	8.50 ± 4.22	0.4
Neutrophils	57.24 ± 21.15	39.40 ± 20.23	56.63 ± 16.48	68.07 ± 14.38	<0.001
Lymphocytes	33.62 ± 20.19	49.81 ± 19.97	37.04 ± 16.67	22.82 ± 12.79	<0.001
Platelets	288.06 ± 114.74	337.67 ± 114.76	253.14 ± 91.58	262.60 ± 109.65	0.019
		Biochemistry Profile			
CRP	27.25 ± 60.43	13.93 ± 49.04	53.62 ± 107.74	28.32 ± 47.63	0.033
ESR	38.07 ± 33.65	43.00 ± 24.04	46.20 ± 46.63	31.75 ± 28.97	0.7
Creatinine	0.45 ± 0.48	0.23 ± 0.14	0.39 ± 0.17	0.61 ± 0.59	<0.001
AST	96.16 ± 290.20	62.00 ± 65.17	93.33 ± 127.88	106.70 ± 350.33	0.3
ALT	107.86 ± 404.48	56.00 ± 109.68	70.33 ± 95.85	138.88 ± 516.76	0.2
GGT	21.88 ± 26.25	23.40 ± 23.16	26.25 ± 34.32	20.31 ± 26.75	>0.9
ALP	184.92 ± 87.42	243.80 ± 88.82	196.75 ± 54.54	162.13 ± 88.48	0.2

^1^ Mean ± SD; ^2^ Kruskal–Wallis rank sum test.

**Table 4:** Treatment, medications, and outcomes across included participants.

Characteristic	Less than 5 Years	6 to 10 Years	11 to 18 Years
	N = 112 ^1^	N = 95 ^1^	N = 278 ^1^
	Respiratory support		
O_2_ support	8 (16%)	1 (2.3%)	3 (3.2%)
HFNC	1 (2.0%)	0 (0%)	0 (0%)
PPV	1 (2.1%)	0 (0%)	0 (0%)
	Medications		
Steroids	6 (13%)	3 (7.7%)	3 (3.6%)
Inotropes	1 (2.0%)	0 (0%)	0 (0%)
Antibiotics	24 (40%)	7 (17%)	31 (30%)
	Outcomes		
Mortality	2 (3.3%)	0 (0%)	0 (0%)
Complications	2 (3.4%)	0 (0%)	0 (0%)

^1^ n (%).

## Data Availability

The data associated with this manuscript are available from the corresponding author upon a reasonable request.

## Supplementary Materials

The following supporting information can be downloaded at: https://www.mdpi.com/article/10.3390/jcm14082577/s1, Table S1: Categorized hematological and biochemistry profiles for included participants stratified by age.
