# Supplementary material for: COVID-19 Infection in Pediatric Patients Presenting to a Tertiary Center in Jordan: Clinical Characteristics and Age-Related Patterns
Source: J Clin Med. 2025 Apr 9;14(8):2577. doi: 10.3390/jcm14082577 (PMC12028193; doi:10.3390/jcm14082577)
Supplement: Supplementary file 1 [file jcm-14-02577-s001.zip › jcm-3505000-supplementary.pdf]

Supplementary Table S1: Categorized hematological and biochemistry profiles for included participants stratified by age.

		Less than 5 years		6 to 10 years		11 to 18 years	
**Variable**	**Category**	**N**	**%**	**N**	**%**	**N**	**%**
WBC	Decreased	0	0.0%	5	11.1%	20	8.7%
	Normal	85	60.7%	20	44.4%	140	60.9%
	Increased	55	39.3%	20	44.4%	70	30.4%
Neutrophils	Decreased	85	63.0%	15	33.3%	0	0.0%
	Normal	50	37.0%	25	55.6%	175	77.8%
	Increased	0	0.0%	5	11.1%	50	22.2%
Lymphocytes	Decreased	15	11.1%	5	12.5%	100	46.5%
	Normal	30	22.2%	20	50.0%	95	44.2%
	Increased	90	66.7%	15	37.5%	20	9.3%
Platelets	Decreased	0	0.0%	10	28.6%	15	7.0%
	Normal	115	85.2%	25	71.4%	185	86.0%
	Increased	20	14.8%	0	0.0%	15	7.0%
Creatinine	Decreased	100	76.9%	15	37.5%	35	17.5%
	Normal	30	23.1%	25	62.5%	155	77.5%
	Increased	0	0.0%	0	0.0%	10	5.0%
AST	Normal	5	20.0%	0	0.0%	0	0.0%
	Increased	20	80.0%	15	100.0%	85	100.0%
ALT	Normal	35	87.5%	10	66.7%	80	94.1%
	Increased	5	12.5%	5	33.3%	5	5.9%
GGT	Decreased	0	0.0%	5	25.0%	5	6.3%
	Normal	15	60.0%	10	50.0%	60	75.0%
	Increased	10	40.0%	5	25.0%	15	18.8%
ALP	Normal	20	80.0%	20	100.0%	70	93.3%
	Increased	5	20.0%	0	0.0%	5	6.7%
CRP	Normal	120	83.3%	24	37.5%	96	44.4%
	Increased	24	16.7%	40	62.5%	120	55.6%
ESR	Normal	0	0.0%	8	20.0%	16	25.0%
	Increased	16	100.0%	32	80.0%	48	75.0%
